# Association of anemia and hemoglobin decrease during acute stroke treatment with infarct growth and clinical outcome

**DOI:** 10.1371/journal.pone.0203535

**Published:** 2018-09-26

**Authors:** Sebastian Bellwald, Rupashani Balasubramaniam, Michael Nagler, Meret S. Burri, Samuel D. A. Fischer, Arsany Hakim, Tomas Dobrocky, Yannan Yu, Fabien Scalzo, Mirjam R. Heldner, Roland Wiest, Marie-Luise Mono, Hakan Sarikya, Marwan El-Koussy, Pasquale Mordasini, Urs Fischer, Gerhard Schroth, Jan Gralla, Heinrich P. Mattle, Marcel Arnold, David Liebeskind, Simon Jung

**Affiliations:** 1 Department of Neurology, Inselspital, Bern University Hospital, University of Bern, Bern, Switzerland; 2 University Institute for Diagnostic and Interventional Neuroradiology, Inselspital, Bern University Hospital, Bern, University of Bern, Switzerland; 3 Department of Haematology and Central Haematology Laboratory, Inselspital, Bern University Hospital, and Department of Clinical Research, University of Bern, Bern, Switzerland; 4 Neurovascular Imaging Research Core and the Department of Neurology, University of California, Los Angeles, United States of America; University of Münster, GERMANY

## Abstract

**Background and purpose:**

Anemia is associated with worse outcome in stroke, but the impact of anemia with intravenous thrombolysis or endovascular therapy has hardly been delineated. The aim of this study was to analyze the role of anemia on infarct evolution and outcome after acute stroke treatment.

**Methods:**

1158 patients from Bern and 321 from Los Angeles were included. Baseline data and 3 months outcome assessed with the modified Rankin Scale were recorded prospectively. Baseline DWI lesion volumes were measured in 345 patients and both baseline and final infarct volumes in 180 patients using CT or MRI. Multivariable and linear regression analysis were used to determine predictors of outcome and infarct growth.

**Results:**

712 patients underwent endovascular treatment and 446 intravenous thrombolysis. Lower hemoglobin at baseline, at 24h, and nadir until day 5 predicted poor outcome (OR 1.150–1.279) and higher mortality (OR 1.131–1.237) independently of treatment. Decrease of hemoglobin after hospital arrival, mainly induced by hemodilution, predicted poor outcome and had a linear association with final infarct volumes and the amount and velocity of infarct growth. Infarcts of patients with newly observed anemia were twice as large as infarcts with normal hemoglobin levels.

**Conclusion:**

Anemia at hospital admission and any hemoglobin decrease during acute stroke treatment affect outcome negatively, probably by enlarging and accelerating infarct growth. Our results indicate that hemodilution has an adverse effect on penumbral evolution. Whether hemoglobin decrease in acute stroke could be avoided and whether this would improve outcome would need to be studied prospectively.

## Introduction

Anemia is a frequently observed pre-existing condition in ischemic stroke patients. A recent meta-analysis of conservatively treated patients indicated a prevalence of 21.9%.[1] According to previous studies, anemia has a negative effect on outcome in conservatively treated patients. [2–6] In addition, two recent meta-analyses including more than 19,000 patients confirmed an increased mortality in such patients with anemia (OR 1.39 respectively 1.97). [1,7] Whether anemia has also an impact in stroke patients treated with intravenous thrombolysis (IVT) or endovascular therapy (EVT), which aim to rescue the penumbral tissue, has hardly been studied. There is only one study on anemia and patients treated with IVT. [8] Anemia was associated with worse outcome, but the impact of anemia in EVT is unknown.

It is a matter of debate, whether the negative association of anemia and stroke outcome is simply based on comorbidities that cause the anemia, or whether decreased oxygen-carrying capacity with anemia accelerates the decay of the penumbra because of reduced oxygen supply to the jeopardized tissue. Two studies described an association of anemia and further hemoglobin decrease with infarct growth. [9,10] They would support the latter mechanism. However, the two studies did not take reperfusion success into account and did not restrict their analysis to patients without anemia at hospital admission. Therefore, a confounding effect of reperfusion and comorbidities cannot be ruled out.

The aim of this study was to analyze the impact of anemia at hospital admission and hemoglobin decrease during treatment on stroke outcome and infarct growth in a large cohort of patients treated with EVT and IVT.

## Patients and methods

### Patients and treatment

The present study includes patients of the Bernese stroke registry, a prospectively collected data base that had been used also for other studies. [11–15] Patients were included in this analysis if: 1) they underwent IVT, EVT or bridging IVT and EVT between January 2011 and June 2015 and 2) at least baseline laboratory parameters were available.

The treating neurologist and neuroradiologist decided whether to perform IVT, EVT or bridging therapy on a case by case basis considering the radiological findings and patient’s baseline characteristics. They comprised age, gender, National Institutes of Health Stroke Scale (NIHSS) score, location of vessel occlusion, time from symptom onset to treatment, atrial fibrillation, hypertension, diabetes, smoking, and hypercholesterolemia. Decision for performing MR or CT at admission was performed on an individual bases. Usually MR was performed in all patients expect for patients with contraindications for MR and patients suffering from vomiting. Reperfusion after EVT was graded by TICI scores. A CT or MRI scan was obtained 24h to 72h after treatment or in any case of clinical deterioration. Symptomatic intracranial hemorrhage (sICH) was graded according to the definition of the PROACT II Study. [16] Clinical outcome was assessed 3 months after the stroke using the modified Rankin Scale (mRS).

Consecutive patients treated at the David Geffen School of Medicine in Los Angeles with IVT or EVT between April 2012 and October 2016 and who had perfusion CT or MR at baseline served as comparison for the amount of hemoglobin decrease after hospital arrival. The data was obtained from a prospective collected data base. Missing parameters were obtained retrospectively if available. The study was performed with approval of the local ethics committees of Bern without the need for written informed consent. All data were anonymized before processing.

### Laboratory parameters

Blood was collected in plastic syringes containing 1.6 ml EDTA per ml of blood (Monovette®, Sarstedt, Nümbrecht, Germany). Samples were analyzed using an automated hematology analyzer (Coulter Counter LH750, Beckman-Coulter Inc., Nyon, Switzerland). Pre-treatment laboratory results of hemoglobin, hematocrit, and erythrocytes and if available, 24h follow up results and nadir until day 5 were collected retrospectively. Anemia was defined according to the World Health Organization criteria as hemoglobin below 12 g/dL in women and below 13 g/dL in men.

### Blood pressure and infusion therapy

In patients in whom segmentation of the final infarct volume was performed, the following additional parameters were collected retrospectively: systolic blood pressure (BP) <120mmHg, systolic BP <110mmHg or BP drop of > 20mmHg within the first 24h and 48h, systolic BP nadir until 48h, infusion volume within 24h and 48h, and use of vasoactive medication.

### Imaging methods and analysis

MRI was performed using a 1.5T or 3T MR imaging system (Siemens Magnetom Avanto/Aera/Verio, Siemens Medical Solution, Erlangen, Germany). The MRI protocol included whole brain diffusion weighted imaging (DWI) (b = 1000t, 24 slices, thickness 5 mm, TR 3200ms, TE 87ms, number of averages 2, matrix 256×256) yielding isotropic b0 and b1000. ADC maps were calculated according to the exponential relation S(b) = S(0) exp(–b • ADC), where S(b) is the signal intensity using diffusion weighting with the value b, and S(0) is the signal intensity with b = 0. Segmentation of the DWI lesion in pre-treatment imaging was performed with the OLEA Sphere Software® (OLEA Medical, La Ciotat, France) by S.B. in all patients who underwent MRI before endovascular treatment. Segmentation of the final infarct was performed with 3D slicer version 4.5.0–1 (manual slice-by-slice delineation with “draw” and “paint” tools by M.B., S.F., controlled by A.H.) in all patients with pre-treatment MRI, who received follow-up imaging by MRI (FLAIR or T2, slice thickness 5mm) or CT (slice thickness 3mm, Siemens Definition Edge, Siemens Healthcare, Erlangen, Germany) 14 days or later after stroke. Baseline and follow-up images were compared before segmentation to exclude the inclusion of infarcts from previous stroke.

### Statistical analysis

SPSS 21 (SPSS Inc., Chicago, Illinois, USA) served for statistical analysis. Bivariable analysis of categorical variables was performed with χ2 and Fisher’s exact test as appropriate and continuous variables with Mann-Whitney test. Because baseline and final infarct volumes and time from symptom onset to treatment were not normally distributed, the natural logarithm-transformed variables were used. Infarct growth velocity was defined as $infarct\;growth\;velocity=\frac{final\;infarct\;volume–baseline\;infarct\;volume}{time\;from\;MRI\;to\;reperfusion}$.

Outcome was dichotomized into favorable (mRS 0–2) or poor clinical outcome (mRS 3–6). Forward stepwise logistic regression analysis including all variables with p<0.2 in bivariate analysis (all baseline characteristics and hemoglobin parameters) was used to determine the predictors of outcome and survival. Linear regression analysis was used to determine the predictors of baseline infarct volume, final infarct volume, infarct growth, and velocity of infarct growth by inclusion of the same variables and, if appropriate, reperfusion success, time from MRI to reperfusion, systolic BP <120mmHg, <110mmHg or BP drop of > 20mmHg within the first 24h and 48h, systolic BP nadir until 48h, infusion volume within 24h and 48h, and use of vasoactive medication. Subgroup analyses were performed for infarct growth and velocity of infarct growth in patients without anemia at baseline. Each laboratory parameter (hemoglobin baseline, 24h, nadir until day 5, hemoglobin decrease) entered separately into the final models to test their significance.

Linear regression analysis was used to determine the predictors of hemoglobin decrease by inclusion of the variables systolic BP nadir, amount of infusion until 24h, time from MRI to reperfusion, and baseline characteristics.

A p-value < 0.05 was considered significant.

The blood loss that equates to hemoglobin decrease after hospital arrival was estimated by the formula $V=EBV\times \ln\left(\frac{hemoglobin\;baseline}{hemoglobin\;nadir}\right)$, where V indicates the volume and EBV the patient’s estimated blood volume. [17] EBV was estimated by multiplying the gender dependent mean body weight of the Swiss population[18] by 65 ml/kg for women and 75 ml/kg for men.

## Results

### Baseline characteristics and laboratory parameters

Baseline characteristics of the 1158 included patients are listed in Table 1. Pre-treatment and follow up laboratory parameters are listed in Table 2. Hemoglobin at admission was available for all 1158 patients, hemoglobin at 24h for 939 patients, and hemoglobin nadir until day 5 for 960 patients. Anemia at hospital admission was observed in 191 (26.8%) of the patients treated with EVT and 92 (20.6%) of the patients treated with IVT. Until day 5, anemia occurred or was unmasked because of fluid replacement in 491/600 (81.8%) of the patients treated with EVT and 181/360 (50.3%) of the patients treated with IVT. Newly observed anemia between hospital arrival and day 5 was noted in 332/600 (46.6%) of the patients treated with EVT and 106/360 (29.4%) of the patients treated with IVT.

**Table 1:** Baseline characteristics and outcome of 1158 treated patients.

	EVT (n = 712)	IVT (n = 446)
Age, years (SD)	71 (13.9)	72.2 (13.6)
Women	352 (49.4%)	172 (38.6%)
**Vascular risk factors**		
- Hypertension	518/710 (73%)	330/444 (74.3%)
- Diabetes mellitus	132/708 (18.6%)	82/444 (18.5%)
- Atrial fibrillation	297/610 (48.7%)	149/394 (37.8%)
- Current smoking	150/578 (26%)	66/386 (17.1%)
- Hypercholesterolemia	400/697 (57.4%)	268/443 (60.5%)
Baseline NIHSS score, median (range)	15 (0–36)	7 (0–36)
**Vessel occlusion location**		
- No occlusion	0	7.6)
- Internal carotid artery	220 (30.9)	5.8)
- Middle cerebral artery	407 (57.2)	0.7)
- Anterior cerebral artery	4 (0.6)	2.7)
- Posterior cerebral artery	14 (2)	6.3)
- Vertebrobasilar system	67 (9.4)	31 (7)
**Treatment**		
- EVT without prior IVT	414/712 (58.1)	-
- Bridging therapy (IVT + EVT)	298/712 (41.9)	-
Minutes from symptom onset to treatment, median (range)	265 (70–1663)	180 (60–1680)
**Outcome**		
- mRS 0–2	253/642 (39.4)	249/398 (62.6)
- Survival	467/642 (72.7)	333/398 (83.7)
**Complications**		
- Symptomatic ICH	32/697 (4.6)	16/428 (3.7)

EVT = endovascular treatment, IVT = intravenous thrombolysis

**Table 2:** Laboratory baseline and follow up parameters (n = 1158 for hemoglobin at admission, n = 939 for hemoglobin at 24h and n = 960 for hemoglobin nadir until day 5).

	EVT (n = 712)	IVT (n = 446)
**Patients treated at University Hospital Bern, Switzerland**		
Hemoglobin baseline, g/dl mean (SD)	13.2 (1.9)	13.7 (1.6)
Hemoglobin 24h, g/dl mean (SD)	11.4 (1.7)	12.8 (1.6)
Hemoglobin nadir until day 5, g/dl mean (SD)	11.1 (1.7)	12.4 (1.7)
Hemoglobin decrease until day 5, g/dl mean (SD)	-2.2 (1.4)	-1.3 (1.1)
Hematocrit baseline, l/l mean (SD)	0.45 (1.49)	0.41 (0.05)
Hematocrit 24h, l/l mean (SD)	0.34 (0.05)	0.38 (0.05)
Hematocrit nadir until day 5, l/l mean (SD)	0.33 (0.05)	0.37 (0.05)
Erythrocytes at baseline, T/l mean (SD)	4.30 (0.58)	4.43 (0.5)
Erythrocytes 24h, T/l mean (SD)	3.74 (0.56)	4.15 (0.52)
Erythrocytes nadir until day 5, T/l mean (SD)	3.62 (0.59)	4.03 (0.56)
**Anemia**		
Anemia at hospital admission	191/712 (26.8)	92/446 (20.6)
Anemia at 24h	444/591 (75.1)	142/348 (40.8)
Anemia until day 5	491/600 (81.8)	181/360 (50.3)
Newly observed anemia until 24h	290/591 (49.1)	80/348 (23)
Newly observed anemia until day 5	332/600 (46.6)	106/360 (29.4)
**Patients treated at David Geffen School Los Angeles, USA**		
Hemoglobin decrease until day 5, g/dl mean (SD)	-2.3 (1.4)	-1.8 (1.3)

EVT = endovascular treatment, IVT = intravenous thrombolysis

### Predictors of 3 months’ outcome and survival

The predictors of outcome, survival and infarct volumes are listed in Table 3. In multivariable regression analysis hemoglobin at baseline, hemoglobin at 24h, hemoglobin nadir until day 5 and hemoglobin decrease until day 5 were independent predictors of outcome. Lower hemoglobin levels or larger decrease were associated with worse outcome. The results were independent from the treatment modality.

**Table 3:** Predictors of outcome and survival in multivariable regression analysis and of infarct volumes and infarct growth in linear regression analysis. For each laboratory parameter, a separate analysis was performed and the results shown for other non-laboratory parameters are based on the model with inclusion of the first listed laboratory parameter. Hemoglobin at admission was available for 1158 patients, hemoglobin at 24h for 939 patients, and hemoglobin nadir until day 5 for 960 patients.

**Predicting factor**	**P**	**OR**	**95% CI**
**mRS 0–2**			
Age	< 0.001	0.945	0.932–0.958
Diabetes mellitus	< 0.001	0.415	0.271–0.635
NIHSS score baseline	< 0.001	0.865	0.843–0.887
Time symptom onset to treatment	< 0.001	0.451	0.317–0.642
sICH	< 0.001	0.034	0.007–0.164
Hemoglobin baseline	0.001	1.177	1.069–1.295
Hemoglobin 24h	0.013	1.150	1.030–1.284
Hemoglobin nadir day 5	< 0.001	1.279	1.155–1.416
Hemoglobin decrease until day 5	0.010	1.193	1.043–1.365
Erythrocytes 24h	0.009	1.559	1.119–2.172
Erythrocytes nadir day 5	<0.001	2.265	1.668–3.076
**Survival**			
Age	< 0.001	0.944	0.929–0.960
Diabetes mellitus	0.01	0.490	0.324–0.741
NIHSS score baseline	< 0.001	0.903	0.883–0.924
sICH	< 0.001	0.080	0.037–0.171
Hemoglobin baseline	< 0.001	1.194	1.081–1.320
Hemoglobin 24h	0.032	1.131	1.010–1.266
Hemoglobin nadir day 5	< 0.001	1.237	1.110–1.379
Erythrocytes 24h	0.002	1.633	1.189–2.244
Erythrocytes nadir day 5	<0.001	2.356	1.758–3.158
**Predicting factor**	**P**	**Regression coefficient**	**T**
**Baseline infarct volume (based on 345 patients)**			
Age	0.001	-0.017	-3.241
Male gender	0.036	-0.323	-2.109
Hemoglobin baseline	0.014	-0.109	-2.475
**Final infarct volume (based on 180 patients)**			
Reperfusion success	0.006	-0.321	-2.788
Systolic BP nadir	0.014	0.022	2.491
Hemoglobin decrease until day 5	0.002	-0.285	-3.146
Newly overserved anemia until day 5	0.023	0.605	2.300
**Infarct growth (based on 180 patients)**			
Reperfusion success	< 0.001	-16.299	-5.267
Hemoglobin decrease until day 5	0.003	-7.596	-3.012
Newly overserved anemia until day 5	0.022	16.964	2.308
**Velocity infarct growth (based on 180 patients)**			
Reperfusion success	< 0.001	-5.026	-3.979
Hemoglobin decrease until day 5	0.001	-3.514	-3.414
Newly observed anemia until day 5	0.016	7.368	2.442

Hemoglobin at baseline, hemoglobin at 24h, and hemoglobin nadir until day 5 were independent predictors of survival.

**Fig 1:**
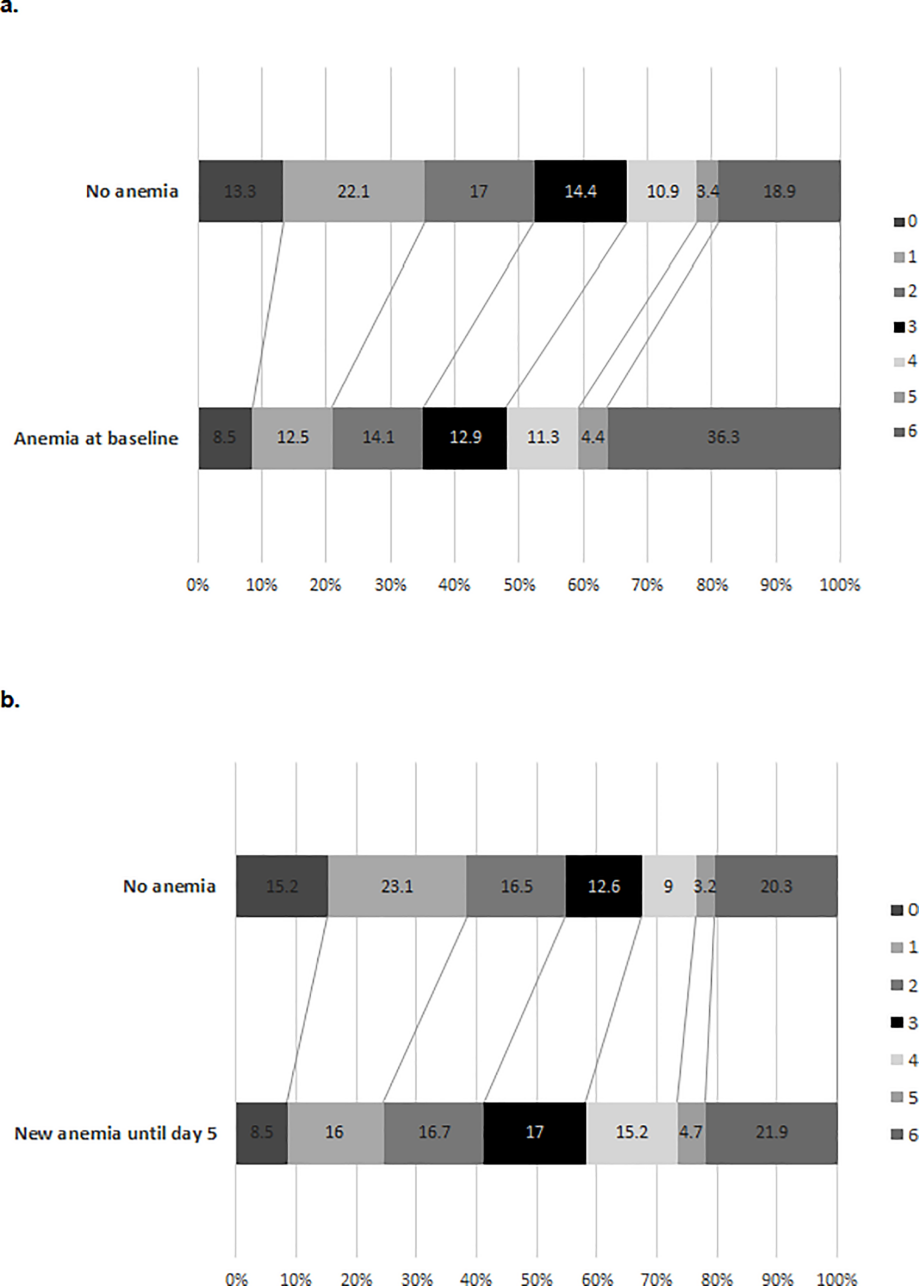
Distribution of 3 months outcome (as measured by modified Rankin Scale score) in dependence on anemia at hospital admission (a) and newly observed anemia until day 5 (b).

The distribution of 3 months’ outcome as measured by modified Rankin Scale score in dependence on baseline anemia and newly observed anemia until day 5 is illustrated in Fig 1. Among 8 patients with newly observed anemia until day 5, one patient achieved a less favorable outcome in our cohort (Number Needed to Harm 7.4).

### Predictors of infarct size and growth

**Fig 2:**
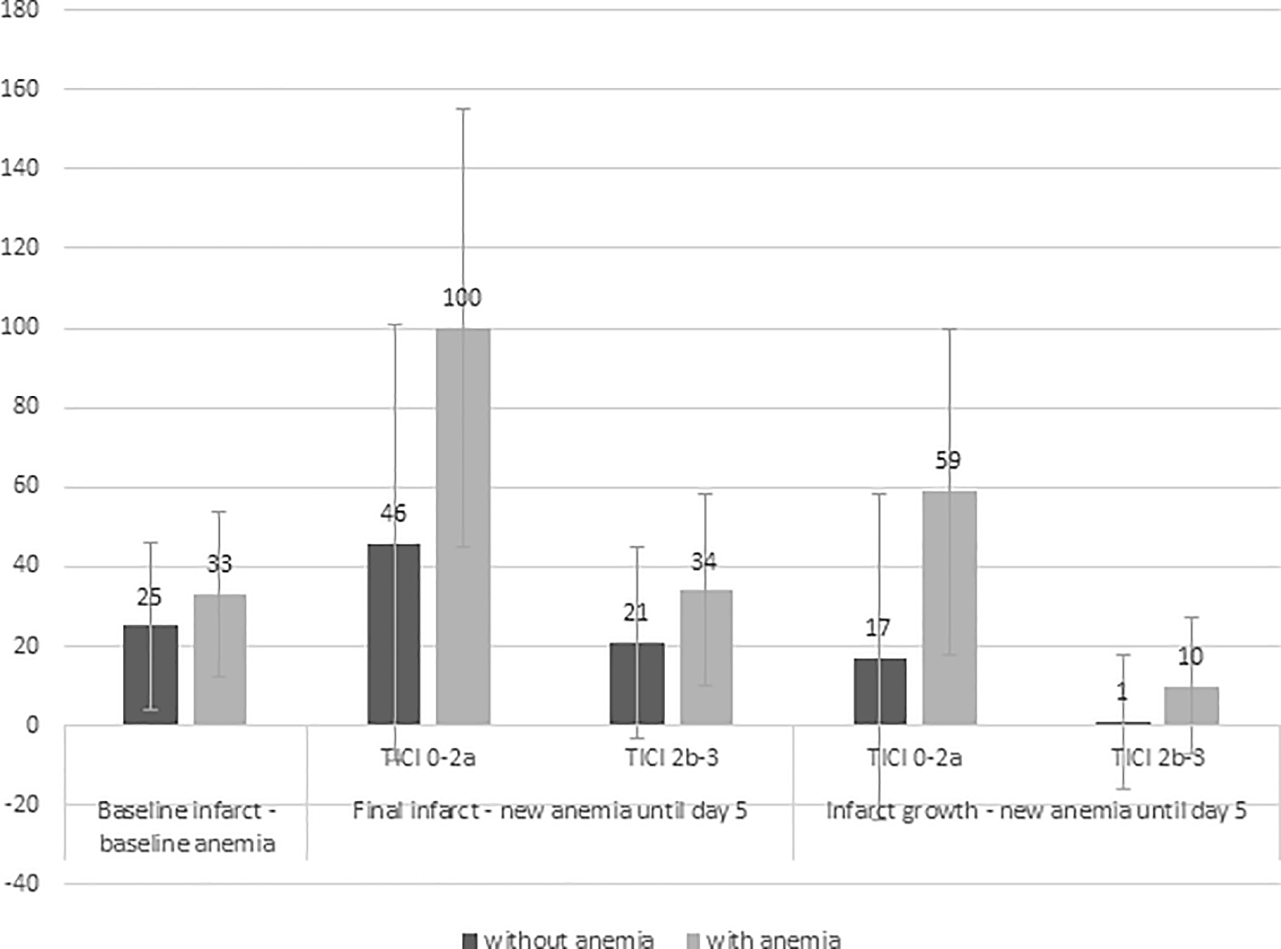
Influence of baseline anemia and newly observed anemia on mean infarct sizes and infarct growth.

Segmentation of MRI was performed in 345 patients treated with EVT to assess baseline infarct volume. Both baseline and final infarct volumes were obtained of 180 patients. Successful reperfusion (TICI score 2b-3) was achieved in 135 (75%) of them. The infarct volumes and velocity of infarct growth in dependence on newly observed anemia until day 5 and reperfusion success are listed in Table 4. The mean infarct volume at baseline was 27 cm^3^ (SD 36) and the final infarct volume 41 cm^3^ (SD 66). Hemoglobin at baseline was an independent predictor of the baseline infarct volume in linear regression analysis (Table 3 and Fig 2). The mean baseline infarct volume in patients without anemia at hospital admission was 25 cm^3^ (SD 34) and 33 cm^3^ (SD 42) in those with anemia at hospital admission. Hemoglobin decrease until 24h was an independent predictor of infarct growth and hemoglobin decrease until day 5 an independent predictor of the final infarct volume and infarct growth (S1 Fig). Hemoglobin decrease until 24h and until day 5 were also independent predictors of the velocity of infarct growth. In subgroup analysis after exclusion of patients with TICI scores 0 or 1, in whom the infarct growth continues after failed recanalization with the endovascular procedure, this result remained robust.

**Table 4:** Association between newly observed anemia and extent of infarction in 180 patients treated with EVT.

	Final infarct volume cm^3^, mean (SD)		Infarct growth cm^3^, mean (SD)		Velocity infarct growth cm^3^/h, mean (SD)	
	without anemia	with anemia	without anemia	with anemia	without anemia	with anemia
**New anemia until day 5**	26 (43)	53 (79)	5 (36)	24 (57)	0.3 (12)	9 (24)
- Reperfusion TICI 0-2a	46 (69)	101 (113)	17 (67)	59 (82)	2.5 (21)	21.5 (36)
- Reperfusion TICI 2b-3	21 (32)	34 (48)	1 (21)	10 (34)	-0.2 (9)	3.2 (13)

The hemoglobin decrease until day 5 proved as predictor of the amount and velocity of infarct growth velocity also in the subgroup of patients with normal hemoglobin at hospital admission. Newly observed anemia until day 5 was an independent predictor of the final infarct volume, infarct growth and infarct growth velocity.

### Predictors of hemoglobin decrease

There was an association between hemoglobin decrease until day 5 and the systolic BP nadir (p = 0.035) and the amount of infusion (p = 0.011) in bivariable analysis. In linear regression analysis, the time from MRI to reperfusion (p = 0.001, regression coefficient -0.006, T = -3.512) and the systolic BP nadir (p = 0.005, regression coefficient 0.020, T = 2.850) were predictors of hemoglobin decrease until day 5. The mean hemoglobin decrease of 2.2 g/dl in patients treated with EVT equates to an estimated amount of blood loss of 890ml, and the decrease of 1.3 g/dl in patients treated with IVT to an estimated amount of 490ml.

### Comparison group of patients treated at the David Geffen School of Medicine in Los Angeles

In patients treated at the David Geffen School of Medicine, Los Angeles, the mean hemoglobin decrease until day 5 was -1.8g/dl (SD 1.3) in 160 patients treated with IVT and -2.3g/dl (SD 1.4) in 161 patients treated with EVT.

## Discussion

The main finding of our study is that anemia at hospital admission and any decrease of hemoglobin are associated with worse outcome and higher mortality in patients treated with IVT or EVT. This finding pertains not only to anemia at hospital admission but also to any hemoglobin decrease thereafter. There was a linear relationship between hemoglobin decrease and larger final infarct volume and greater and faster infarct growth. The reason of the relationship of hemoglobin and outcome is unknown, but a causal relationship between lower hemoglobin and faster infarct growth is conceivable. Anemia entails a decrease of oxygen-carrying capacity of blood, however confounding effects like overused fluid replacement during the acute recanalization and reperfusion therapy or thereafter may contribute partly to this association. Excessive hemodilution might impact negatively on the penumbra and optimal fluid replacement in acute stroke would need to be addressed in further studies.

Several trials found a negative impact of anemia at hospital admission on outcome and survival in conservatively treated ischemic stroke patients. [1–7, 19] The impact of anemia in acute stroke treatment with IVT or EVT has been addressed in only one study of 217 patients treated with IVT, which found a negative impact of anemia on outcome and survival. [8] In our cohort of 1158 patients, anemia at hospital admission and lower hemoglobin at any time during the first 5 days after stroke turned out as a predictor of worse outcome and higher mortality, independently from the treatment modality. In addition, a larger hemoglobin decrease after hospital arrival was associated with worse outcome. The potential effect size, expressed as number needed to harm for suffering unfavorable outcome due to newly observed anemia would be 7.4. However, this effect size has to be interpreted with caution because our data are not based on a randomized trial.

The reason for the negative effect of anemia on outcome is unknown. Two studies describing an association of anemia with infarct growth assumed that decreased oxygen-carrying capacity with anemia might exert a negative effect on penumbra evolution. [9,10] Our study confirms their assumption and provides additional information on the relationships of hemoglobin, reperfusion and infarct growth. In our study, there was not only a linear relationship between lower hemoglobin at hospital arrival and larger baseline infarct volumes, but also a linear relationship between hemoglobin decrease after hospital arrival and greater and faster infarct growth (S1 Fig). Hemoglobin decrease after hospital arrival turned out as the only predictor of infarct growth in addition to reperfusion failure. A similar effect was observed even in patients in whom hemoglobin did not fall to the level of anemia definition. This indicates that even small changes of hemoglobin concentration might jeopardize the optimal oxygen supply to the penumbra. The hemoglobin decrease after hospital admission resulted likely from hemodilution, because there was a linear association between hemoglobin decrease and amount of infusion therapy and because stroke therapy does not result in significant blood loss. The hemoglobin decrease of patients treated at the University Hospital of Bern and patients treated at the David Geffen School in Los Angeles were similar which rules out a center specific overuse of infusion therapy.

Our findings raise the question whether we need to rethink current infusion therapy for stroke patients. Does infusion therapy account for worse outcome or do confounding factors explain the accelerated infarct growth with decreasing hemoglobin? First, low BP was associated with hemoglobin decrease in our cohort and may have contributed to infarct growth by itself. Second, longer time from CT or MRI to reperfusion turned out as predictor of hemoglobin decrease, which may stand for longer infusion times and amounts because of more complicated and time-consuming endovascular procedures. Third, patients with larger infarcts are usually more severely affected, less likely able to swallow and more likely to receive intravenous fluid replacement. Although part of the infarct growth may be explained by these three factors, hemoglobin decrease turned out as a predictor of infarct growth independently from blood pressure, time to successful or failed reperfusion and infarct severity. In addition, animal studies showing larger infarcts after hemodilution support a causal relationship of hemodilution with infarct growth. [20–22] A model based on in vitro data predicts a continuous decline of oxygen delivery in the penumbra already below hemoglobin levels of 10g/dl. [23] Assuming no confounding factors and based on the regression coefficient for infarct growth, infusion of 1000 ml NaCl 0.9% might result in infarct growth up to 3.1ml or 7% of the mean final infarct volume. [24–26] That hemodilution should have a negative impact on infarct size might be somehow unexpected, because hemodilution was believed to improve outcome by enhancing blood viscosity in earlier days and only randomized trials disproved this concept. [27] According to the formula Q × Hb × SaO2 × 1.39 (where Q indicates blood flow and SaO2 arterial oxygen saturation), that describes oxygen delivery to the brain, any decrease of hemoglobin may affect oxygen delivery when it is not compensated by an increased blood flow. [28] In healthy volunteers, raised cardiac output and NO induced vasodilatation can increase cerebral blood flow and keep oxygen delivery stable down to hemoglobin levels of 5–6 g/dl. [29,30] However, in the ischemic penumbra all compensatory mechanisms are exhausted and already at their limits. [31,32] To summarize, a negative impact of even a small hemoglobin decrease induced by hemodilution on infarct growth in acute stroke is conceivable. It needs a closer look and further studies should investigate, whether and how it should and could be avoided.

The most important limitation of our study is its retrospective character. Therefore, we cannot exclude confounding effects that are at least partly responsible for the association of hemoglobin decrease with infarct growth. A further limitation is the accuracy of the determination of the final infarct volume. It may have been influenced by the techniques of follow up imaging (CT and MRI).

## Supporting information

S1 Dataset hemoglobin_data.sav. (SAV)

S1 Fig Adjusted predicted final infarct volumes in dependence on hemoglobin decrease until day 5, calculated by linear regression analysis by inclusion of factors reperfusion success and systolic blood pressure nadir. (TIF)

## References

[1] Li Z, Zhou T, Li Y, Chen P, Chen L. Anemia increases the mortality risk in patients with stroke: A meta-analysis of cohort studies. Sci Rep. Nature Publishing Group; 2016;6: 26636 10.1038/srep26636 27211606 PMC4876389

[2] Kimberly WT, Lima FO, O’Connor S, Furie KL. Sex differences and hemoglobin levels in relation to stroke outcomes. Neurology. 2013;80: 719–24. 10.1212/WNL.0b013e31828250ff 23365064 PMC3589294

[3] Kellert L, Schrader F, Ringleb P, Steiner T, Bösel J. The impact of low hemoglobin levels and transfusion on critical care patients with severe ischemic stroke. STroke: RelevAnt Impact of HemoGlobin, Hematocrit and Transfusion (STRAIGHT)-an observational study. J Crit Care. Elsevier Inc.; 2014;29: 236–240. 10.1016/j.jcrc.2013.11.008 24332995

[4] Park YH, Kim BJ, Kim JS, Yang MH, Jang MS, Kim N, et al Impact of both ends of the hemoglobin range on clinical outcomes in acute ischemic stroke. Stroke. 2013;44: 3220–3222. 10.1161/STROKEAHA.113.002672 24003047

[5] Lasek-Bal A1, Holecki M2, Stęposz A3 DJ. The impact of anemia on the course and short-term prognosis in patients with first ever ischemic stroke. Neurol Neurochir Pol. 2015; 107–12. 10.1016/j.pjnns.2015.03.001 25890925

[6] Sico JJ1, Concato J, Wells CK, Lo AC, Nadeau SE, Williams LS, Peixoto AJ, Gorman M, Boice JL BD. Anemia is associated with poor outcomes in patients with less severe ischemic stroke. J Stroke Cerebrovasc Dis. 2013; 271–8. 10.1016/j.jstrokecerebrovasdis.2011.09.003 22100828

[7] Barlas RS, Honney K, Loke YK, McCall SJ, Bettencourt‐Silva JH, Clark AB, et al Impact of Hemoglobin Levels and Anemia on Mortality in Acute Stroke: Analysis of UK Regional Registry Data, Systematic Review, and Meta‐Analysis. J Am Heart Assoc. 2016;5: e003019 10.1161/JAHA.115.003019 27534421 PMC5015269

[8] Kellert L, Martin E, Sykora M, Bauer H, Gussmann P, Diedler J, et al Cerebral oxygen transport failure?: Decreasing hemoglobin and hematocrit levels after ischemic stroke predict poor outcome and mortality: Stroke: relevant impact of hemoglobin, hematocrit and transfusion (STRAIGHT)—An observational study. Stroke. 2011;42: 2832–2837. 10.1161/STROKEAHA.110.606665 21852622

[9] Kellert L, Herweh C, Sykora M, Gussmann P, Martin E, Ringleb PA, et al Loss of Penumbra by Impaired Oxygen Supply? Decreasing Hemoglobin Levels Predict Infarct Growth after Acute Ischemic Stroke: Stroke: Relevant Impact of Hemoglobin, Hematocrit and Transfusion (STRAIGHT)—An Observational Study. Cerebrovasc Dis Extra. 2012;2: 99–107. 10.1159/000343731 23599701 PMC3567874

[10] Kimberly WT, Wu O, Arsava EM, Garg P, Ji R, Vangel M, et al Lower hemoglobin correlates with larger stroke volumes in acute ischemic stroke. Cerebrovasc Dis Extra. 2011;1: 44–53. 10.1159/000328219 22566982 PMC3343751

[11] Luedi R, Hsieh K, Slezak A, El-Koussy M, Fischer U, Heldner MR, et al Age dependency of safety and outcome of endovascular therapy for acute stroke. J Neurol. 2014;261: 1622–7. 10.1007/s00415-014-7401-0 24916832

[12] Jung S, Gilgen M, Slotboom J, El-Koussy M, Zubler C, Kiefer C, et al Factors that determine penumbral tissue loss in acute ischaemic stroke. Brain. 2013;136: 3554–60. 10.1093/brain/awt246 24065722

[13] Gilgen MD, Klimek D, Liesirova KT, Meisterernst J, Klinger-Gratz PP, Schroth G, et al Younger Stroke Patients with Large Pretreatment Diffusion-Weighted Imaging Lesions May Benefit from Endovascular Treatment. Stroke. 2015;46: 2510–2516. 10.1161/STROKEAHA.115.010250 26251252

[14] Jung S, Mono ML, Fischer U, Galimanis A, Findling O, De Marchis GM, et al Three-month and long-term outcomes and their predictors in acute basilar artery occlusion treated with intra-arterial thrombolysis. Stroke. 2011/05/07. 2011;42: 1946–1951. 10.1161/STROKEAHA.110.606038 21546481

[15] Jung S, Schindler K, Findling O, Mono ML, Fischer U, Gralla J, et al Adverse effect of early epileptic seizures in patients receiving endovascular therapy for acute stroke. Stroke. 2012/04/27. 2012;43: 1584–1590. 10.1161/STROKEAHA.111.645358 22535264

[16] Kase CS, Furlan a J, Wechsler LR, Higashida RT, Rowley H a, Hart RG, et al Cerebral hemorrhage after intra-arterial thrombolysis for ischemic stroke: the PROACT II trial. Neurology. 2001;57: 1603–10. Available: http://www.ncbi.nlm.nih.gov/pubmed/11706099 11706099 10.1212/wnl.57.9.1603

[17] Gross J. Estimating Allowable Blood Loss Corrected for Dilution. Anesthesiology. 1983;58: 277–280. 6829965 10.1097/00000542-198303000-00016

[18] Average body weight of the Swiss population [Internet]. Available: https://www.worlddata.info/average-bodyheight.php

[19] Hatamian H, Saberi A, Pourghasem M. The relationship between stroke mortality and red blood cell parameters. Iran J Neurol. 2014;13: 237–40. Available: http://www.ncbi.nlm.nih.gov/pubmed/25632337 25632337 PMC4300800

[20] Homi HM, Yang H, Pearlstein RD, Grocott HP. Hemodilution during cardiopulmonary bypass increases cerebral infarct volume after middle cerebral artery occlusion in rats. Anesth Analg. 2004;99: 974–981. 10.1213/01.ANE.0000131504.90754.D0 15385336

[21] Karadibak K, Gökmen N, Erbayraktar S, Göktay Y, Taplu A, Arkan A, et al Effects of normovolaemic haemodilution on middle cerebral artery blood flow velocity and oxygen delivery. Eur J Anaesthesiol. 2002;19: 330–6. Available: http://www.ncbi.nlm.nih.gov/pubmed/12095012 12095012 10.1017/s0265021502000534

[22] Reasoner DK, Ryu KH, Hindman BJ, Cutkomp J, Smith T. Marked hemodilution increases neurologic injury after focal cerebral ischemia in rabbits. Anesth Analg. 1996;82: 61–7. Available: http://www.ncbi.nlm.nih.gov/pubmed/8712427 8712427 10.1097/00000539-199601000-00011

[23] Dexter F, Hindman BJ. Effect of haemoglobin concentration on brain oxygenation in focal stroke: a mathematical modelling study. Br J Anaesth. 1997;79: 346–351. Available: http://eutils.ncbi.nlm.nih.gov/entrez/eutils/elink.fcgi?dbfrom=pubmed&id=9389854&retmode=ref&cmd=prlinks%5Cnpapers2://publication/uuid/A84F9F8D-6D06-40D5-9A61-7F85C5A7457D 9389854 10.1093/bja/79.3.346

[24] Lobo DN, Stanga Z, Simpson J a, Anderson J a, Rowlands BJ, Allison SP. Dilution and redistribution effects of rapid 2-litre infusions of 0.9% (w/v) saline and 5% (w/v) dextrose on haematological parameters and serum biochemistry in normal subjects: a double-blind crossover study. Clin Sci (Lond). 2001;101: 173–9. 10.1042/CS20000316 11473492

[25] De Aguilar-Nascimento JE, Valente AC, Oliveira SS, Hartmann A, Slhessarenko N. Changes in body composition, hematologic parameters, and serum biochemistry after rapid intravenous infusion or oral intake of 2 liters of 0.9% saline solution in young healthy volunteers: Randomized crossover study. World J Surg. 2012;36: 2776–2781. 10.1007/s00268-012-1756-0 22948196

[26] Grathwohl KW, Bruns BJ, LeBrun CJ, Ohno AK, Dillard TA, Cushner HM. Does hemodilution exist? Effects of saline infusion on hematologic parameters in euvolemic subjects. South Med J. 1996;89: 51–5. Available: http://www.ncbi.nlm.nih.gov/pubmed/8545692 8545692 10.1097/00007611-199601000-00008

[27] Chang TS, Jensen MB. Haemodilution for acute ischaemic stroke. Cochrane database Syst Rev. 2014; CD000103 10.1002/14651858.CD000103.pub2 25159027 PMC4181849

[28] Lelubre C, Bouzat P, Crippa IA, Taccone FS. Anemia management after acute brain injury. Crit Care. Critical Care; 2016;20: 152 10.1186/s13054-016-1321-6 27311626 PMC4911680

[29] Weiskopf RB, Viele MK, Feiner J, Kelley S, Lieberman J, Noorani M, et al Human cardiovascular and metabolic response to acute, severe isovolemic anemia. JAMA. 1998;279: 217–21. Available: http://www.ncbi.nlm.nih.gov/pubmed/9438742 9438742 10.1001/jama.279.3.217

[30] Weiskopf RB, Kramer JH, Viele M, Neumann M, Feiner JR, Watson JJ, et al Acute severe isovolemic anemia impairs cognitive function and memory in humans. Anesthesiology. 2000;92: 1646–52. Available: http://www.ncbi.nlm.nih.gov/pubmed/10839915 10839915 10.1097/00000542-200006000-00023

[31] Aries MJH, Elting JW, De Keyser J, Kremer BPH, Vroomen PCAJ. Cerebral Autoregulation in Stroke: A Review of Transcranial Doppler Studies. Stroke. 2010;41: 2697–2704. 10.1161/STROKEAHA.110.594168 20930158

[32] Atkins ER, Brodie FG, Rafelt SE, Panerai RB, Robinson TG. Dynamic Cerebral Autoregulation Is Compromised Acutely following Mild Ischaemic Stroke but Not Transient Ischaemic Attack. Cerebrovasc Dis. 2010;29: 228–235. 10.1159/000267845 20029195

