# Supplementary figures and images for: Association of anemia and hemoglobin decrease during acute stroke treatment with infarct growth and clinical outcome
Source: PLoS One. 2018 Sep 26;13(9):e0203535. doi: 10.1371/journal.pone.0203535 (PMC6157859; doi:10.1371/journal.pone.0203535)

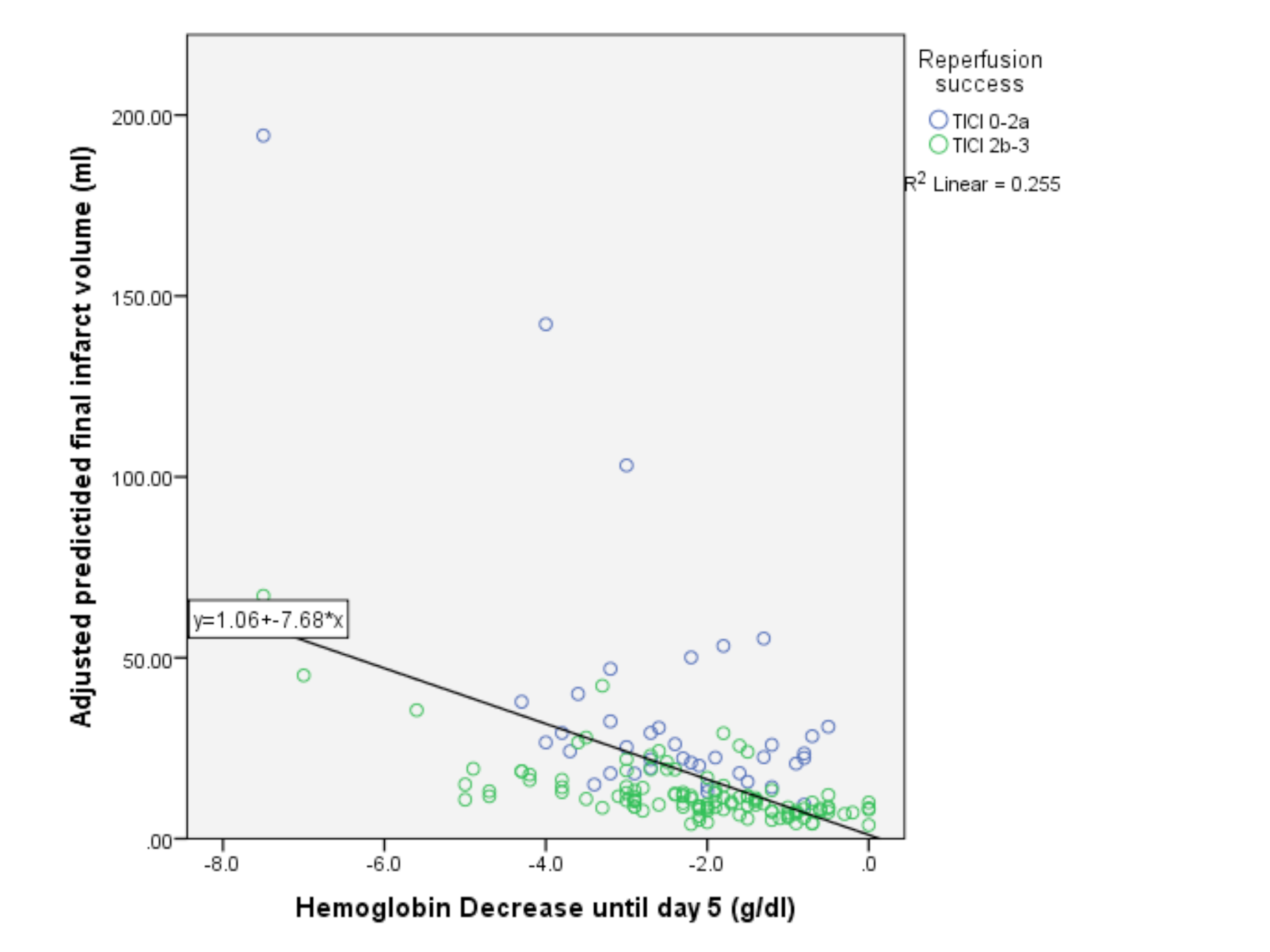

Supplement: S1 Fig — (TIF) [file pone.0203535.s002.tif]
